# Anatomy and evolution of the pectoral filaments of threadfins (Polynemidae)

**DOI:** 10.1038/s41598-020-74896-y

**Published:** 2020-10-20

**Authors:** Paulo Presti, G. David Johnson, Aléssio Datovo

**Affiliations:** 1 Museu de Zoologia da Universidade de São Paulo, Avenida Nazaré, 481, Ipiranga, São Paulo, SP 04263-000 Brazil; 2 National Museum of Natural History, Smithsonian Institution, Washington, DC USA

**Keywords:** Ichthyology, Evolution

## Abstract

The most remarkable anatomical specialization of threadfins (Percomorphacea: Polynemidae) is the division of their pectoral fin into an upper, unmodified fin and a lower portion with rays highly modified into specialized filaments. Such filaments are usually elongate, free from interradial membrane, and move independently from the unmodified fin to explore the environment. The evolution of the pectoral filaments involved several morphological modifications herein detailed for the first time. The posterior articular facet of the coracoid greatly expands anteroventrally during development. Similar expansions occur in pectoral radials 3 and 4, with the former usually acquiring indentations with the surrounding bones and losing association with both rays and filaments. Whereas most percomorphs typically have four or five muscles serving the pectoral fin, adult polynemids have up to 11 independent divisions in the intrinsic pectoral musculature. The main *adductor* and *abductor* muscles masses of the pectoral system are completely divided into two muscle segments, each independently serving the pectoral-fin rays (dorsally) and the pectoral filaments (ventrally). Based on the innervation pattern and the discovery of terminal buds in the external surface of the filaments, we demonstrate for the first time that the pectoral filaments of threadfins have both tactile and gustatory functions.

## Introduction

Polynemids are easily identifiable as a natural group based on their external morphology, particularly their distinct pectoral fin divided into a dorsal part, with 12–19 soft rays united by an interradial membrane, and a ventral portion with around 3–16 isolated rays that are usually elongated, forming filaments with tactile functions^1,2^.

Most previous morphological studies of polynemids have largely focused on superficial analyses of specific structures, such as vertebrae and pectoral-filament counts^1,3,4^, caudal fin anatomy^5^ and some cephalic bones^6,7^. With the exception of the study of Kang et al.^8^, detailed osteological descriptions (e.g., jaws, neurocranium and pectoral girdle) are available only for a few new genera and/or species^6,9–11^. Information on polynemid myology and neurology is even more limited. Gosline^7^ superficially described the *adductor mandibulae* of *Polydactylus octonemus*, and Springer and Johnson^12^ described the dorsal gill-arch muscles of *Polydactylus oligodon* and *Filimanus xanthonema.* Kang et al.^8^ presented a more comprehensive study of polynemid morphology, but their anatomical descriptions are somewhat superficial and most skeletal muscles and nerves were set aside from the analysis. A few generalized aspects of the innervation in a polynemid was provided by Freihofer^13^. Only more recently, Presti et al.^14^ provided detailed descriptions and illustrations of the facial and gill-arch musculature of several polynemids.

The goals of the present study are to investigate in detail the modifications that have occurred in the skeleton, muscles and nerves associated with the appearance of the specialized polynemid pectoral filaments. For this purpose, we examined representatives of all polynemid genera, including an ontogenetic series of one of these species, and compared it with the anatomy of other percomorphs proposed to be variously related to the family based on morphology.

## Material and methods

This study was carried out under approval of the Animal Care and Use Committee (ACUC) of the Instituto de Biociências, Universidade de São Paulo to A. Datovo (Project #226/2015; CIAEP #01.0165.2014). The research employed only ethanol-preserved specimens deposited in museums and did not involve animal experimentation or fossil examination. Examined specimens are from the following institutions: Academy of Natural Sciences of Drexel University, USA (ANSP); California Academy of Sciences, USA (CAS); Natural History Museum of Los Angeles County, USA (LACM); and Museu de Zoologia da Universidade de São Paulo, Brazil (MZUSP). In total, 25 adult specimens representing 19 polynemid species were double stained for analysis of their skeleton, musculature, and innervation: *Eleutheronema tetradactylum* (1) MZUSP 123253, (1) ANSP 61928; *Eleutheronema tridactylum* (1) ANSP 89554; *Filimanus similis* (1) MZUSP 124826; *Filimanus xanthonema* (1) MZUSP 123255; *Galeoides decadactylus* (1) MZUSP 123256; *Leptomelanosoma indicum* (1) CAS 50925; *Parapolynemus verekeri* (1) MZUSP 123718; *Pentanemus quinquarius* (2) MZUSP 123254; *Polydactylus approximans*, (1) MZUSP 124822; *Polydactylus microstomus* (1) MZUSP 124823; *Polydactylus octonemus* (1) MZUSP 124817; *Polydactylus oligodon* (1) MZUSP 67533; *Polydactylus opercularis* (1) MZUSP 124825; *Polydactylus plebeius* (1) MZUSP 124824; *Polydactylus sexfilis* (1) MZUSP 124812; *Polydactylus sextarius* (1) MZUSP 124811; *Polydactylus virginicus* (3) MZUSP 51249, (1) MZUSP 67546; *Polynemus multifilis* (1) MZUSP 63339; *Polynemus paradiseus* (2) MZUSP 123252^14^. Additionally we examined an ontogenetic series of *Polydactylus approximans* (LACM 9789-2), ranging from 4 to 21.3 mm standard length (SL). Comparative material: Sciaenidae: *Bairdiella ronchus* (1) MZUSP 112,337; *Cynoscion striatus* (2) MZUSP 6891. Sphyraenidae: *Sphyraena tome* (1) MZUSP 47,562. Mugilidae: *Mugil curema* (1) MZUSP 67314^14^. Access to material of these collections and permission for dissections were duly authorized by the respective curators.

For the study of the cephalic and pectoral fin musculoskeletal system and innervation pattern of adults, specimens were double stained following the protocol of Datovo and Bockmann^15^. For the ontogenetic study, larval and juvenile specimens of *Polydactylus approximans* were cleared and double stained for the study of bones and cartilages following the techniques of Taylor and Van Dyke^16^ and Schnell et al.^17^. All specimens were at the post-flexion stage and were measured from the anterior end of the premaxilla to the posterior portion of the hypurals (standard length, SL). Osteological terminology followed primarily Hilton et al.^18^, with the following adaptations: autosphenotic for the ossification preformed in cartilage, with the term sphenotic retained for the compound bone formed by the fusion of the autosphenotic and dermosphenotic; and postcoracoid process for the posteriorly directed projection in the coracoid or scapulocoracoid cartilage^19,20^. Myological nomenclature followed Winterbottom^21^, with necessary additional adaptations justified in the body of the text. Terminology for the nerves followed primarily Freihofer^13^.

Photographs were taken from dissected specimens in order to accelerate the illustration process. Images from larger structures were taken via digital camera Nikon D7000 whereas small structure images were obtained via digital camera Zeiss Axiocam 506 Color attached to a stereomicroscope Zeiss SteREO Discovery.V20. X-ray microcomputed tomography (µCT-scan) images were obtained from a microtomography Phoenix v|tome|x m microfocus of General Electric Company. The image reconstructions were done by Phoenix datos|× 2 reconstruction; GE Sensing and Inspection Technologies GmbH and edited via VG Studio Max version 2.2.3.69611 64bits, Volume Graphics GmbH. Anatomical drawings were based on photographs and direct stereomicroscopic observations of specimens in order to capture fine anatomical details. The black and white illustrations were hand drawn with graphite, while digital bidimensional colour drawings were produced with a Wacom Intuos Comic pen tablet. Outlines were generated in Adobe Illustrator CC 2015 and the shading and colouring in Adobe Photoshop CC 2015.

## Results

### Skeleton and development (Figs. 1, 2)

Early in development, polynemid larvae have a pectoral-girdle and fin morphology comparable to that of generalized percomorphs, that is, with a pectoral fin undivided and lacking differentiated filaments. Drastic ontogenetic changes in the pectoral rays, pectoral proximal radials, and coracoid are primarily responsible for the differentiation of the pectoral fin of adult polynemids. We were able to study these changes in an ontogenetic series of *Polydactylus approximans*, as described below.

In earlier ontogenetic stages, a single cartilaginous pectoral radial plate is present and, at approximately 4 mm SL, this plate is already subdivided into a dorsal and a ventral subplate. At around 4.6 mm SL the dorsal subplate starts to subdivide to form each pectoral radials 1 and 2 cartilage, whereas the ventral subplate will form radials 3 and 4 remains undivided. The scapulocoracoid is fully cartilaginous and has an elongate and posteriorly directed postcoracoid process. No trace of ossification and differentiation in the pectoral-fin rays is observed in these stages.

**Figure 1:**
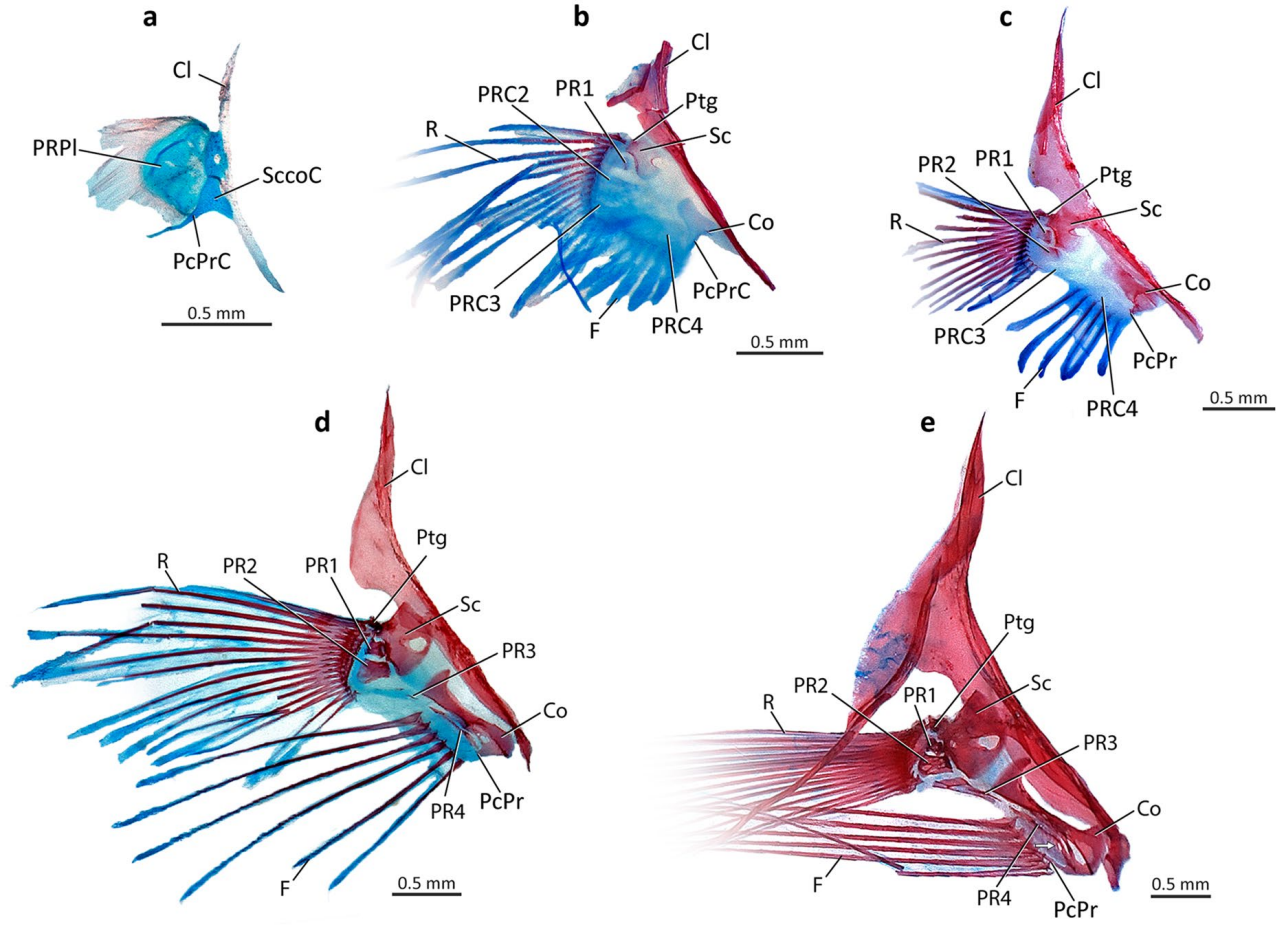
Medial views of cleared and stained ontogenetic series of left pectoral girdle in *Polydactylus approximans,* Polynemidae; (**a**) 4.6 mm SL; (**b**) 8.0 mm SL; (**c**) 8.9 mm SL; (**d**) 13.3 mm SL; (**e**) 21.3 mm SL. Cl, cleithrum; Co, coracoid; F, pectoral filaments; PcPr, postcoracoid process; PcPrC, postcoracoid process cartilage; PR1–4, pectoral radial 1–4; PRC1–4, pectoral radial cartilage 1–4; PRPl, pectoral radial plate; Ptg, propterygium; R, pectoral-fin rays; Sc, scapula; SccoC, scapulocoracoid cartilage. White arrow indicates the foramen on pectoral radial 4. Blue staining on pectoral-fin rays and filaments likely due to affinity of Alcian Blue with collagen rather than indicative of presence of cartilaginous tissue.

At approximately 8 mm SL, all four pectoral-radial cartilages are distinguishable from one another, and only the first one has started to ossify proximally (Fig. 1b). Pectoral radial 3 starts to tilt and shift from horizontal to a more vertical position, and pectoral radial 4 starts to enlarge ventrally. The coracoid shows the first signs of ossification on the posteroventral region of the scapulocoracoid cartilage, anterior to the postcoracoid process cartilage. Although rays and filaments are still contiguous with each other at this stage, a regionalization into a dorsal set of rays and ventral set of early filaments is evident, marking the beginning of the differentiation of the pectoral fin. The pectoral-fin rays have begun to ossify and articulate with pectoral radials 1 to 3. Ossification in these rays occurs from the dorsal to the ventral rays and from the proximal to the distal region of each ray. At this stage, the early pectoral filaments consist of bundles of actinotrichia lacking any associated ossification (lepidotrichia). These early filaments are considerably thicker and shorter than the ossifying pectoral rays and articulate primarily with pectoral radial 4.

**Figure 2:**
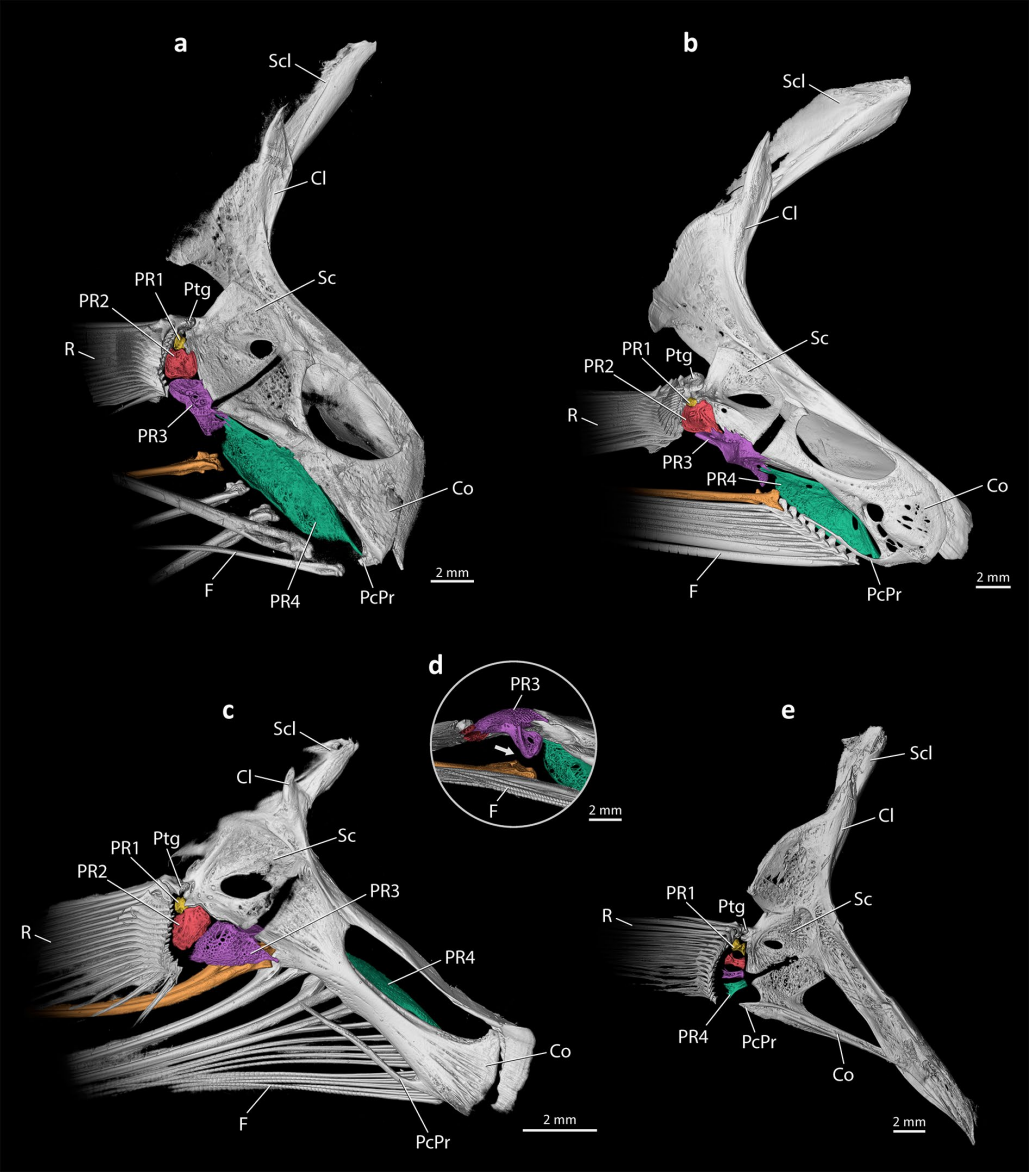
X-ray microcomputed tomographies of left pectoral girdle in medial view; (**a**) *Pentanemus quinquarius*, Polynemidae; (**b**) *Polydactylus opercularis*, Polynemidae; (**c**) *Polynemus multifilis*, Polynemidae; (**d**) ventral view of pectoral radial 3 of *P. multifilis*, Polynemidae; (**e**) *Cynoscion striatus*, Sciaenidae. Cl, cleithrum; Co, coracoid; F, pectoral filaments; PcPr; postcoracoid process; PR1–4, pectoral radial 1–4; Ptg, propterygium; R, pectoral-fin rays; Sc, scapula; Scl, supracleithrum. Arrow indicates site of articulation between pectoral radial 3 and the dorsalmost pectoral filament in (**d**); postcoracoid process broken distally in (**a**).

At 8.9 mm SL almost the whole body of the first pectoral radial is ossified, while the second one has started to ossify at the anterodorsal region of its cartilage (Fig. 1c). The coracoid ossification is well developed, with the postcoracoid process starting to ossify (Fig. 1c). Pectoral-fin rays and pectoral filaments have further diverged from each other and are now separated by a considerable gap. At this stage, all but the two ventralmost pectoral-fin rays are well ossified. The basal portions of all the pectoral filaments are ossified, following a generalized ossification pattern from dorsal to ventral and from proximal to distal.

At 13.3 mm SL the first pectoral radial cartilages is fully ossified, whereas the second one is almost reaching the same stage (Fig. 1d). The third and the fourth pectoral radials start to ossify at their proximal portions. Pectoral radial 3 still articulates with the ventralmost pectoral-fin rays. The coracoid is well ossified (Fig. 1d), except for the postcoracoid process. At this stage the ossified portions of the pectoral filaments have nearly the same length as the ossified portions of the pectoral-fin rays.

At 17.5 mm SL the second pectoral radial is fully ossified, while the two ventralmost radials are ossified along half of their extents (not shown). At this size, the third radial no longer supports any pectoral-fin rays. The foramen in pectoral radial 4 is formed at 17.9 mm SL. At 21.3 mm SL all pectoral-fin rays and pectoral filaments are fully ossified, and the coracoid has reached the adult condition (Fig. 1e). At 32.8 mm SL, the third and fourth pectoral radials are fully ossified.

These ontogenetic changes result in the highly specialized osteology of the pectoral complex of adult polynemids. All adult polynemids have the first two radials articulating with the unmodified pectoral-fin rays, while an enlarged pectoral radial 4 supports the pectoral filaments (Figs. 1e, 2a–c). Pectoral radial 3 does not articulate with any ray or filament, with two exceptions: in *Polynemus* it articulates with the dorsalmost pectoral filament (Fig. 2c, d), and in *Pentanemus* it articulates with the ventralmost unmodified rays (Fig. 2a). In all adult polynemids, most of the pectoral filaments are thicker than the unmodified fin rays, showing a different pattern of segmentation. While the pectoral-fin rays present a roughly uniform pattern of segmentation along each ray, the pectoral filaments have shortened segments proximately that gradually increase in length towards their tips. There is a considerable variation in the number, thickness, and notably the maximum length of the pectoral filaments, which vary from nearly the same size as the unmodified pectoral-fin rays (*Eleutheronema* and *Galeoides*) to roughly three times the total body length (*Polynemus kapuasensis*^1^).

### Lateral musculature (Figs. 3, 4)

**Figure 3:**
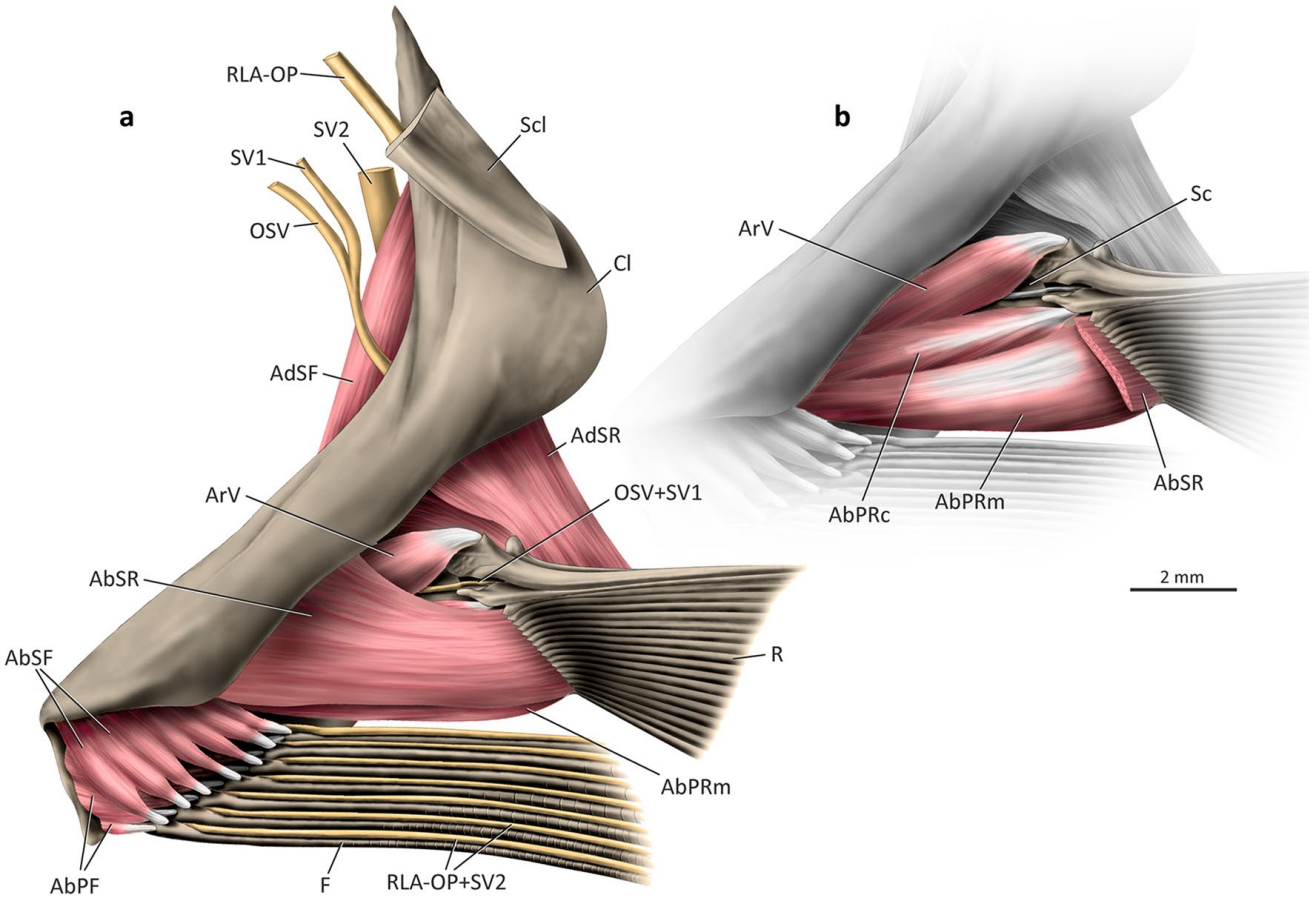
Lateral view of left pectoral girdle of *Polydactylus virginicus*, Polynemidae. (**a**) superficial layers of muscles; (**b**) deepest layers of muscles (AbSR cut and removed). AbPF, *abductor profundus filamentaris*; AbPRc, *abductor profundus radialis p. ceterae*; AbPRm, *abductor profundus radialis p. marginalis*; AbSF, *abductor superficialis filamentaris*; AbSR, *abductor superficialis radialis*; AdSF, *adductor superficialis filamentaris*; AdSR, *adductor superficialis radialis*; ArV, *arrector ventralis*; Cl, cleithrum; F, pectoral filaments; OSV, compound ventral ramus of occipito-spinal nerves; R, pectoral-fin rays; RLA-OP, orbito-pectoralis branch of the *ramus lateralis accessorius*; Sc, scapula; Scl, supracleithrum; SV1–2, ventral *ramus* of spinal nerves 1–2.

In all adult polynemids the *abductor* muscle mass serving the pectoral fin is divided into two independent segments herein named *segmentum radii* (genitive of Latin *radius*, meaning ray) and *segmentum fili* (genitive of Latin *filum*, meaning thread or filament). The former is responsible for moving the unmodified fin rays, while the latter serves the pectoral filaments (Fig. 3).

**Figure 4:**
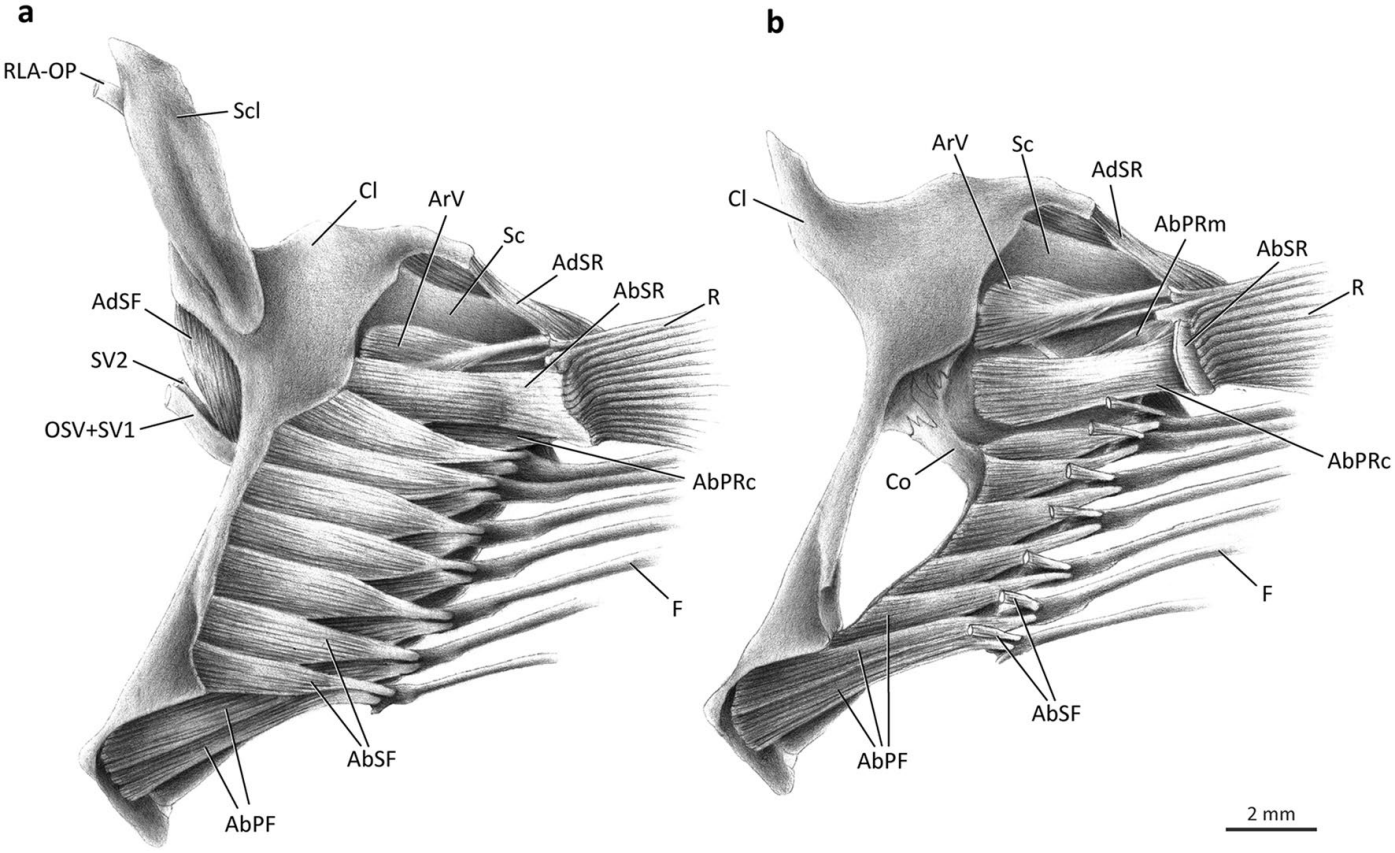
Lateral view of left pectoral girdle of *Parapolynemus verekeri*, Polynemidae. (**a**) superficial layers of muscles; (**b**) deepest layers of muscles (AbSR, AbSF, OSV, Scl, RLA-OP, SV1, and SV2 removed). AbPF, *abductor profundus filamentaris*; AbPRc, *abductor profundus radialis p. ceterae*; AbPRm, *abductor profundus radialis p. marginalis*; AbSF, *abductor superficialis filamentaris*; AbSR, *abductor superficialis radialis*; AdSF, *adductor superficialis filamentaris*; AdSR, *adductor superficialis radialis*; ArV, *arrector ventralis*; Cl, cleithrum; Co, coracoid; F, pectoral filaments; OSV, compound ventral ramus of occipito-spinal nerves; R, pectoral-fin rays; RLA-OP, orbito-pectoralis branch of the *ramus lateralis accessorius*; Sc, scapula; Scl, supracleithrum; SV1–2, ventral *ramus* of spinal nerves 1–2.

The *segmentum radii* of the *abductor superficialis* (= *abductor superficialis radii*; AbSR) is usually the largest lateral muscle component of the unmodified fin, forming a thick unit of fibers at the lateral surface of the horizontal arm of the cleithrum (Fig. 3a). Towards insertion, the muscle gradually differentiates into small bundles that serve each individual ray, although this differentiation is not complete. In most polynemids, the *abductor superficialis radii* originates mainly musculously from the concave surface of the lateral projection of the cleithrum and inserts tendinously on the bases of the lateral hemitrichia of the pectoral-fin rays, except for the first (= marginal) ray. *Parapolynemus* is unique in having the *abductor superficialis radii* originating entirely from the lateral surface of the coracoid (Fig. 4a). Insertion of the muscle is conservative amongst all polynemid genera. The dorsomedial surface of the muscle is aponeurotic and contacts a similarly aponeurotic area of the *adductor profundus radii* (see below).

The *abductor profundus radii* (= *segmentum radii* of the *abductor profundus*; AbPR) is located directly beneath the *abductor superficialis radii* and is completely divided into two sections (Fig. 3b, wherein AbSR is cut and largely removed). The first section, herein termed *pars marginalis* (genitive of Latin *marginalis* meaning marginal; AbPRm), is a bipinnate muscle that originates from the concave surface of the lateral projection of the cleithrum and coracoid and inserts via an elongate tendon on the posteroventral flange of the lateral base of the marginal (= first) ray (Fig. 3b). The second division of the *abductor profundus radii* serves all pectoral-fin rays except the first one and is accordingly named *pars ceterae* (genitive of Latin *ceterus* meaning others, remainder, rest; AbPRc). This muscle section is a single unit at its origin and progressively differentiates into bundles that attach tendinously to each individual ray, although some intermingling of fibers between the bundles may occur (Figs. 3b, 4b). The *partes marginalis* and *ceterae* are completely separate in all examined members of the family, except *Pentanemus* in which there is only superficial differentiation. The origin of the *pars ceterae* is musculous and usually associated with the concave surface of the lateral projection of the cleithrum, coracoid, and third pectoral radial (Figs. 3b, 4b). The scapula is additionally involved in the muscle origin in *Eleutheronema*, *Filimanus*, *Pentanemus,* and most *Polydactylus* (except *P. microstomus, P. sexfilis,* and *P. sextarius*). The *pars ceterae* of *Parapolynemus* originates only from the coracoid and pectoral radial 3 (Fig. 4b). The dorsalmost portion of the *pars ceterae* of the *abductor profundus radii* is more elongate and covered by a lateral aponeurosis (Fig. 3b). The *pars marginalis* of the *abductor profundus radii* is a bipinnate muscle partially covered by the *pars ceterae* and runs parallel to both this section and the *arrector ventralis* (Fig. 3b). *Parapolynemus* is exceptional in having a *pars marginalis* not bipinnate and positioned in an oblique angle relative to the adjacent muscles (Fig. 4b). Most polynemids have a *pars marginalis* originating primarily from the lateral surfaces of the cleithrum, coracoid, and scapula; in *Galeoides* and *Leptomelanosoma* it additionally originates from the lateral face of the third pectoral radial. *Parapolynemus* has the *pars marginalis* originating only from the coracoid and scapula.

The *arrector ventralis* (ArV) is a long, bipinnate muscle that forms the dorsalmost muscle component serving the lateral portion of the pectoral fin (Figs. 3, 4). Most of the muscle is located medial to the *segmentum radii* of the *abductores superficialis* and *profundus*. It originates musculously from the lateral surface of the cleithrum, coracoid and scapula; insertion is via an elongate tendon on the enlarged medial base of the first ray (Fig. 3b).

The *segmentum fili* of the *abductor superficialis* (= *abductor superficialis fili*; AbSF) is usually located on the ventral portion of the girdle. Origin is musculous and primarily from the lateral projection of the cleithrum, where the dorsalmost fibers are overlapped by the ventral portion of the *abductor superficialis radii* (Fig. 3a). *Parapolynemus*, however, has the opposite condition: the *segmentum fili* covers laterally the ventralmost fibers of the *segmentum radii* (Fig. 4a). The *abductor superficialis fili* inserts tendinously on the dorsal region of the base of the lateral hemitrichia of all filaments. In most polynemids, the *segmentum radii* is larger than the *segmentum fili* (Fig. 3a), but in *Parapolynemus*, *Pentanemus*, and *Polynemus* the opposite is true (Fig. 4). The fibers of the *abductor superficialis fili* are clustered into bundles that serve each filament individually. The degree of differentiation of these bundles varies considerably across the family. In *Parapolynemus*, *Pentanemus*, and *Polynemus* they are well-developed and well-separated from each other (Fig. 4), while there is continuity in the remaining polynemids, especially at the muscle origin (Fig. 3a).

The *segmentum fili* of the *abductor profundus* (= *abductor profundus fili*; AbPF) is medial to the same segment of the *abductor superficialis*, originating musculously from the lateral faces of the cleithrum and coracoid (Fig. 3a). In *Galeoides* the fibers additionally arise from the lateral surface of the fourth pectoral radial. Insertion of the *abductor profundus fili* in all polynemids is invariably tendinous onto the lateral base of each filament. The muscle is also differentiated into bundles corresponding to the number of filaments. The dorsal bundle usually overlaps part of the subsequent ventral ones, except in *Galeoides* and *Parapolynemus*. As seen in the *abductor superficialis fili*, the bundles of the *abductor profundus fili* of *Parapolynemus*, *Pentanemus*, and *Polynemus* are well developed and separated from each other compared to other genera (Fig. 4b). The fibers of the *segmentum fili* of the *abductores superficialis* and *profundus* are usually oriented differently: the former muscle has a posteroventral orientation towards the insertion, whereas the latter is oriented posterodorsally (Figs. 3a, 4).

### Medial musculature (Figs. 5–7)

**Figure 5:**
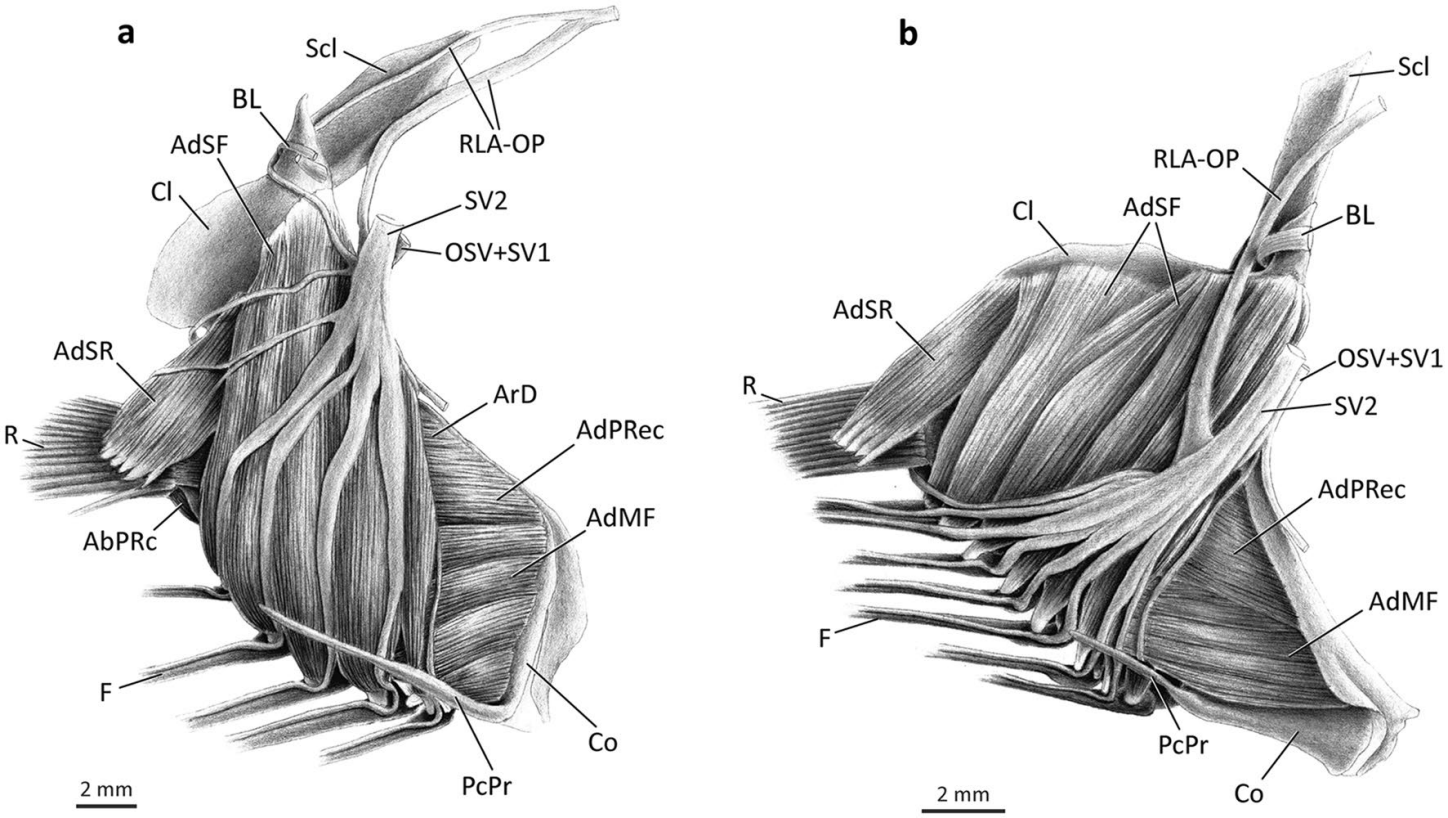
Medial view of left pectoral girdle. (**a**) *Pentanemus quinquarius*, Polynemidae; (**b**) *Parapolynemus verekeri*, Polynemidae. AbPRc, *abductor profundus radialis p. ceterae*; AbSR, *abductor superficialis radialis*; AdMF, *adductor medialis filamentaris*; AdPRec, *adductor profundus radialis p. ectoprofunda*; AdSF, *adductor superficialis filamentaris*; AdSR, *adductor superficialis radialis*; ArD, *arrector dorsalis*; BL, Baudelot’s ligament; Cl, cleithrum; Co, coracoid; F, pectoral filaments; OSV, compound ventral ramus of occipito-spinal nerves; PcPr, postcoracoid process; R, pectoral-fin rays; RLA-OP, orbito-pectoralis branch of the *ramus lateralis accessorius*; Scl, supracleithrum; SV1–2, ventral *ramus* of spinal nerves 1–2.

**Figure 6:**
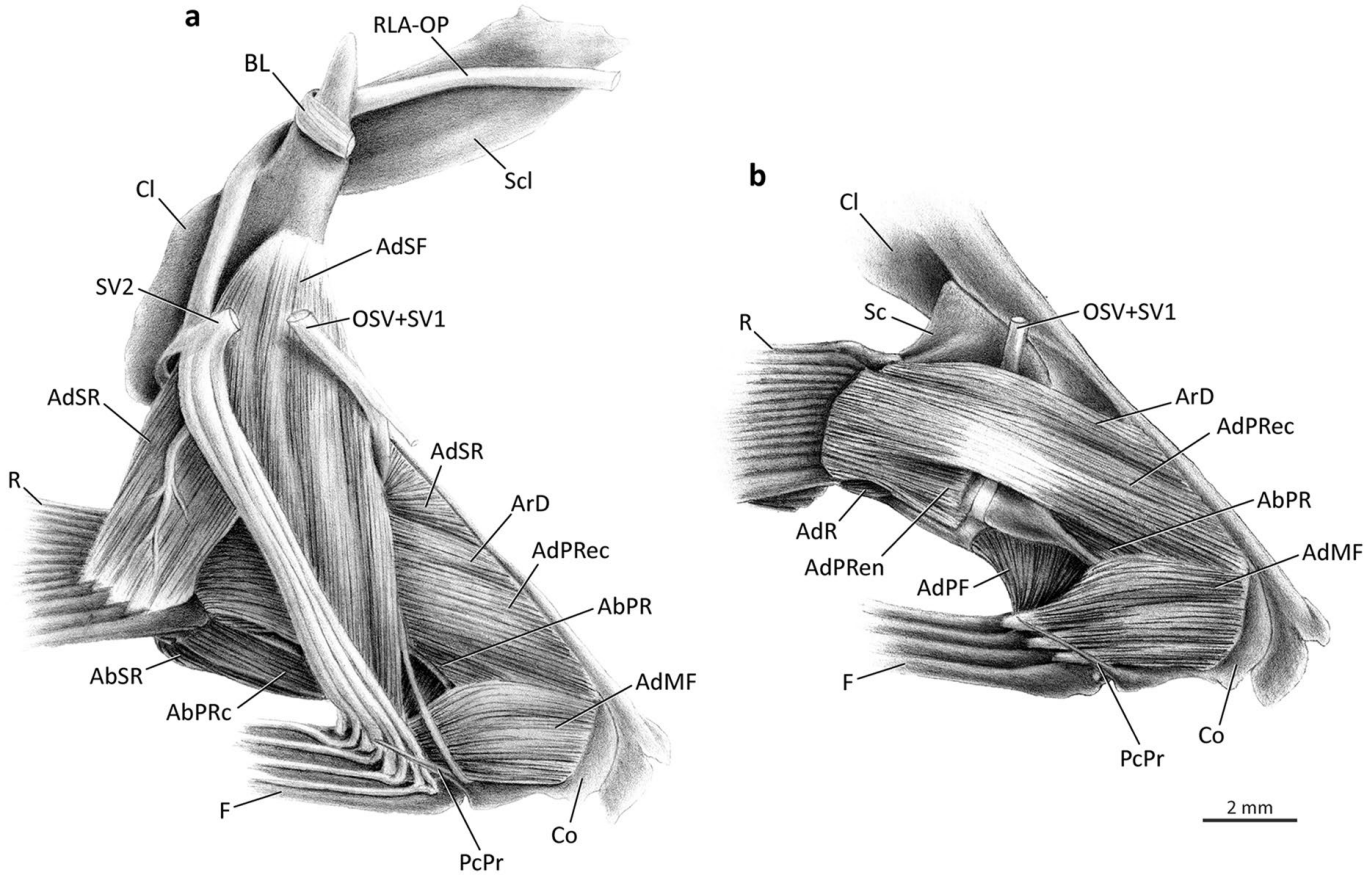
Medial view of left pectoral girdle of *Eleutheronema tetradactylum*, Polynemidae. (**a**) superficial layers of muscles; (**b**) deepest layers of muscles (AdSR, AdSF, RLA-OP, and SV2 removed). AbPRc, *abductor profundus radialis p. ceterae*; AbSR, *abductor superficialis radialis*; AdMF, *adductor medialis filamentaris*; AdPF, *adductor profundus filamentaris*; AdPRec, *adductor profundus radialis p. ectoprofunda*; AdPRen, *adductor profundus radialis p. endoprofunda*; AdR, *adductor radialis*; AdSF, *adductor superficialis filamentaris*; AdSR, *adductor superficialis radialis*; ArD, *arrector dorsalis*; BL, Baudelot’s ligament; Cl, cleithrum; Co, coracoid; F, pectoral filaments; OSV, compound ventral ramus of occipito-spinal nerves; PcPr, postcoracoid process; R, pectoral-fin rays; RLA-OP, orbito-pectoralis branch of the *ramus lateralis accessorius*; Sc, scapula; Scl, supracleithrum; SV1–2, ventral *ramus* of spinal nerves 1–2.

As in the case with the *abductores* of the pectoral fin, the *adductor* muscle mass is also divided into *segmenta radii* and *fili* that serve the unmodified fin rays and the pectoral filaments, respectively (Fig. 5). The *segmentum radii* of the *adductor superficialis* (= *adductor superficialis radii*; AdSR) is a U-shaped well-developed muscle (Fig. 6a) that extends beyond the posterior margin of the cleithrum, being thus partially visible in lateral view just above the bases of the pectoral-fin rays (Figs. 3, 4). Its origin is usually from the medial surface of the cleithrum and scapula. The *adductor superficialis radii* of *Galeoides* and *Leptomelanosoma* additionally arises from the coracoid. The origin of the muscle in most polynemids is mixed, with the dorsalmost fibers having an aponeurotic origin and the ventralmost arising musculously. *Galeoides, Polydactylus microstomus*, and *P. sextarius* are exceptional in having the *adductor superficialis* originating entirely musculously from the cleithrum and scapula. The muscle inserts via tendons on the medial region of the rays, relatively distal from their bases. Except the first ray that is served by the *arrector dorsalis* (see below), all remaining rays are served by the *adductor superficialis radii*. The fibers serving the ventral rays have a progressively more dorsal origin (Fig. 6a). The fibers associated with the second and third rays are much more prominent than the remaining fibers and have a well-developed tendinous portion, giving these portions a nearly bipinnate aspect. Nevertheless, all polynemids lack clear subdivision within the *adductor superficialis radii*.

In polynemids an *adductor medialis radii* is not distinguishable from the surrounding medial muscles. The *adductor profundus radii* is further differentiated into *partes ectoprofunda* (AdPRec) and *endoprofunda* (AdPRen; Fig. 6b). The *pars ectoprofunda* is a robust layer of fibers that originates from the anterior margin of the cleithrum, medial face of the coracoid, and also from the membrane that covers the fenestra between these two bones (Fig. 6b). The *pars endoprofunda* is a deep, thin layer of shorter fibers originating only from the posterodorsal end of the medial face of the coracoid (Fig. 6b). Some fibers may also arise from the medial face of the scapula and from the adjoining area between this bone and the coracoid. As the fibers proceed to their insertion, the *partes ectoprofunda* and *endoprofunda* gradually merge, and the entire *adductor profundus radii* inserts on the medial base of all medial hemitrichia (Fig. 6b).

The *arrector dorsalis* (ArD) is not fully separated from the *adductor profundus radii*, with the two muscles sharing some fibers proximate to their attachment on the pectoral girdle (Figs. 5, 6). Close to the tendinous insertion, fibers of each muscle become more distinguishable from one another. Most fibers corresponding to the *arrector dorsalis* originate musculously from the medial faces of the cleithrum and coracoid and insert on the medial projection of the medial hemitrichium of the first (marginal) ray.

An *adductor radialis* (AdR) is also present and it shares fibers with the *adductor profundus radii* (Fig. 6b). The relatively short fibers of the *adductor radialis* originate musculously primarily from the medial surface of the first three pectoral radials and the medial face of the scapula and insert tendinously on the medial hemitrichium of the ventralmost rays.

**Figure 7:**
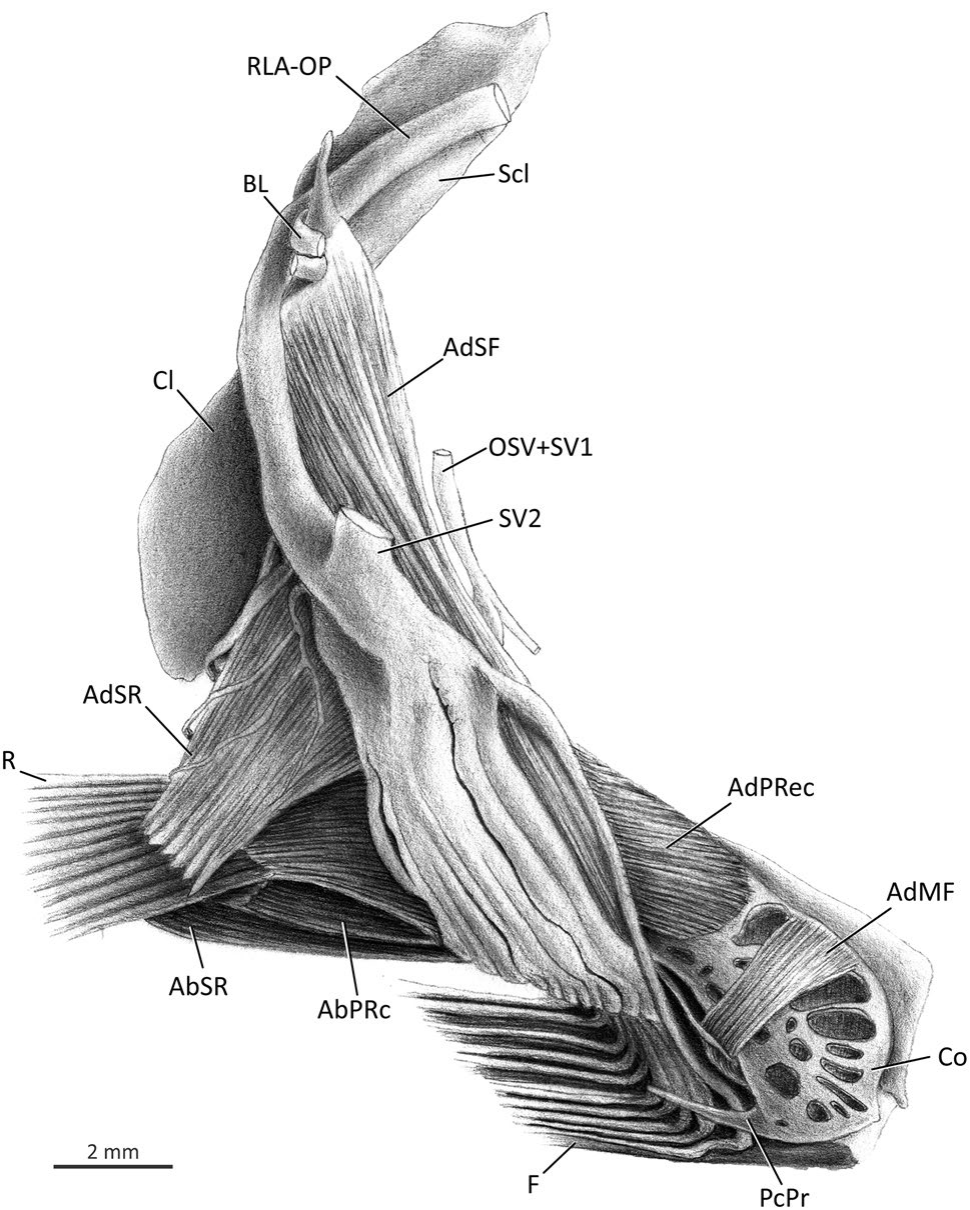
Medial view of left pectoral girdle of *Galeoides decadactylus*, Polynemidae. AbPRc, *abductor profundus radialis p. ceterae*; AbSR, *abductor superficialis radialis*; AdMF, *adductor medialis filamentaris*; AdPRec, *adductor profundus radialis p. ectoprofunda*; AdSF, *adductor superficialis filamentaris*; AdSR, *adductor superficialis radialis*; BL, Baudelot’s ligament; Cl, cleithrum; Co, coracoid; F, pectoral filaments; OSV, compound ventral ramus of occipito-spinal nerves; PcPr, postcoracoid process; R, pectoral-fin rays; RLA-OP, orbito-pectoralis branch of the *ramus lateralis accessorius*; Sc, scapula; Scl, supracleithrum; SV1–2, ventral *ramus* of spinal nerves 1–2.

The *segmentum fili*, in medial view, is divided into *adductores superficialis, medialis,* and *profundus* (Figs. 5 and 7), with some variation detected in the *adductor medialis* (see below). The *adductor superficialis fili* (= *segmentum fili* of the *adductor superficialis*; AdSF) is the major and most superficial muscle component of the medial face of the pectoral girdle (Figs. 5, 6a, 7). Part of this muscle covers the origin of the *adductor superficialis radii*. The *adductor superficialis fili* is subdivided into well-differentiated bundles that serve each individual filament (Figs. 5, 6a, 7). The muscle originates tendinously from the dorsomedial face of the cleithrum and inserts, also via tendons, onto the medial hemitrichia of each pectoral filament. The anteriormost fibers are visible in lateral view, anterior to the cleithrum (Figs. 3, 4a). Origin of the muscle is usually immediately ventral to Baudelot’s ligament (Figs. 5b, 7), but in *Eleutheronema, Polydactylus plebeius,* and *P. sexfilis* the *adductor superficialis fili* originates more ventrally on the medial face of the cleithrum (Fig. 6a). The thickness of the muscle bundles serving each individual filament varies among genera and, the longer the filament, the thicker the bundle is.

The *segmentum fili* of the *adductor medialis* (= *adductor medialis fili*; AdMF) is located in between the same segments of the *adductores superficialis* and *profundus*. The fibers of the *adductor medialis fili* are more horizontal than those of the two other muscles, which have fibers in a more vertical disposition (Figs. 5–7). None of these muscles share fibers. The *adductor medialis fili* is further differentiated into a lateral, innermost subsection termed *pars endomedialis*, with shorter and thinner bundles, and a more superficial, prominent subsection named *pars ectomedialis*, with longer and thicker bundles. As the fibers proceed towards insertion, these subsections gradually merge and are no longer separable from each other. Both subsections of the *adductor medialis fili* originate musculously from the medial surface of the coracoid and insert tendinously on the medial hemitrichia of the filaments. The muscle of *Parapolynemus* and *Polynemus* additionally originates from the cleithrum (Fig. 5b); in *Parapolynemus* the fibers arise from the medial surface of that bone, and in *Polynemus* a few dorsal fibers also arise from the lateral surface of the cleithrum, passing through the cleithrum-coracoid fenestra.

Insertion of the *adductor medialis fili* is quite variable among polynemids, with the muscle either (i) attaching on all pectoral filaments, a condition present in *Filimanus*, *Polydactylus microstomus, P. octonemus, P. oligodon, P. virginicus*, and *Polynemus*; (ii) reaching all but the ventralmost filament in *Eleutheronema*, *Leptomelanosoma*, *Pentanemus*, *Polydactylus approximans, P. opercularis, P. plebeius,* and *P. sexfilis* (Figs. 5a, 6); (iii) reaching all but the two ventralmost filaments in *Parapolynemus* (Fig. 5b); or (iv) attaching only to three of ten filaments in *Galeoides* (Fig. 7).

Most polynemid genera have the *adductor medialis fili* proportionally developed according to the length of their respective pectoral filament. *Galeoides* is an exception, having a poorly developed muscle with no differentiation into *partes endomedialis* and *ectomedialis* and leaving most of its highly perforated coracoid exposed in medial view (Fig. 7). *Eleutheronema* and *Polydactylus sexfilis* have an undivided *adductor medialis fili*, thus differing from the condition of other polynemids in which this muscle is subdivided into individual bundles usually serving each pectoral filament. These muscle bundles are either partially continuous with each other—the most common condition across polynemids—or fully divided, as in *Parapolynemus, Pentanemus,* and *Polynemus*. *Parapolynemus* has seven pectoral filaments and only five bundles of the *adductor medialis fili* (Fig. 5b). Among these, only four bundles differentiate into *partes endomedialis* and *ectomedialis*; the ventralmost bundle, which inserts on the third pectoral filament, is a single, undifferentiated muscle mass. The *pars ectomedialis* of *Polynemus* is divided into three large bundles that insert only on the three dorsalmost pectoral filaments, whereas the *pars endomedialis* serves all filaments. The fibers of the *pars endomedialis* of *Polynemus* additionally arise from the medial face of the fourth pectoral radial. In *Pentanemus* all four bundles have both the *partes endomedialis* and *ectomedialis* serving the four pectoral filaments.

The *segmentum fili* of the *adductor profundus* (= *adductor profundus fili*; AdPF) is the lateralmost muscle of the medial portion of the pectoral girdle (Fig. 6b). Its origin is musculous mainly from the medial surface of the fourth pectoral radial (which is greatly expanded in polynemids), with fibers also arising from the posteroventral margin of the medial face of the coracoid and from the medial surface of the third radial. Usually, only the posterior fibers of the *adductor profundus fili* (i.e*.* those inserting on the uppermost filament) arise from the third radial. Insertion is tendinous on the medial hemitrichia of all filaments. The *adductor profundus fili* is usually differentiated into bundles that serve each individual filament and has a fan-like aspect, with a broad origin and a narrower insertion (Fig. 6b).

### Innervation (Figs. 5–8)

**Figure 8:**
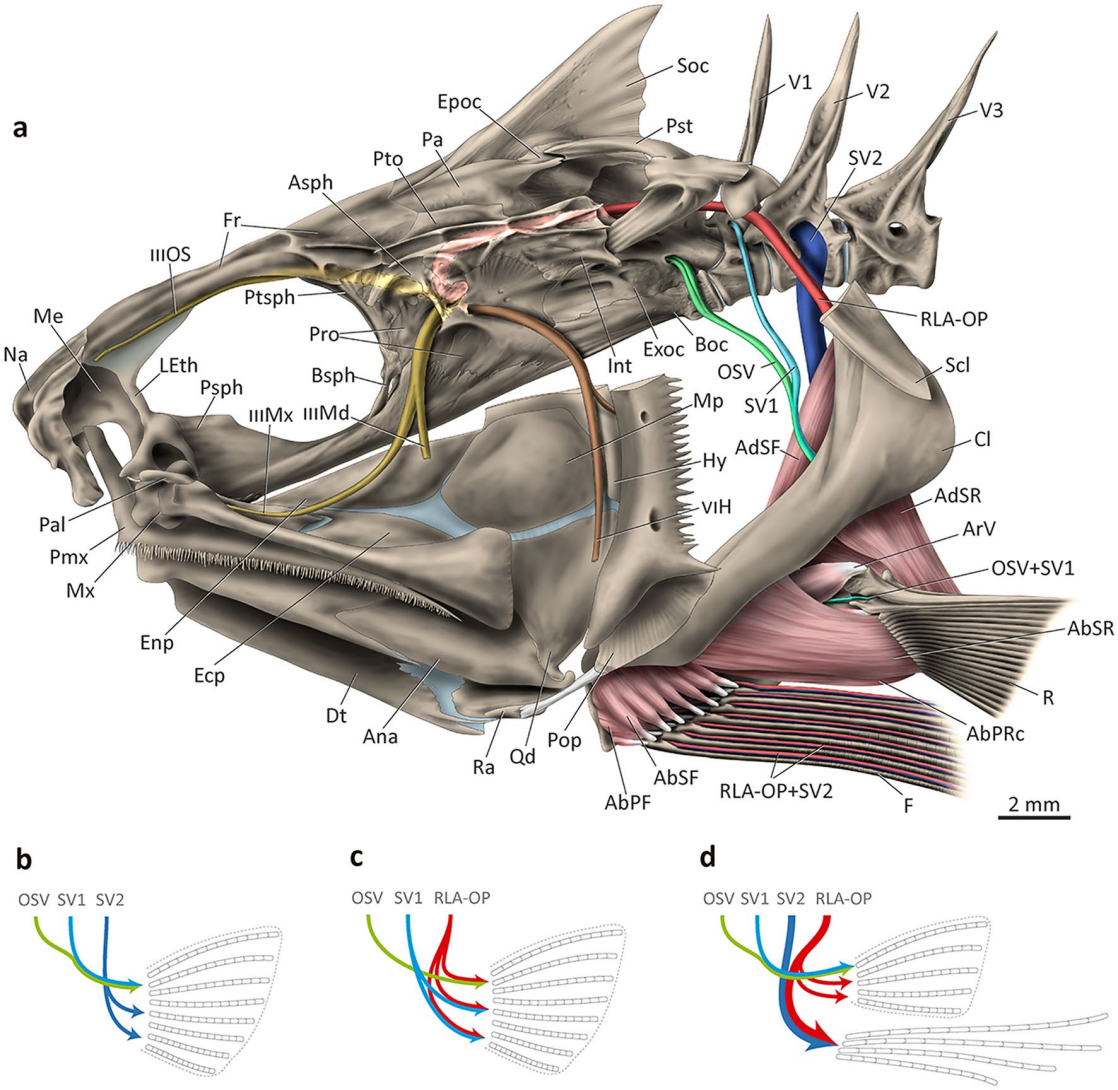
Innervation patterns of pectoral-fin rays of percomorphs. (a) Left lateral view of cranio-pectoral skeleton of *Polydactylus virginicus*, Polynemidae; opercle, subopercle, interopercle, and dorsal portions of hyomandibula and preopercle not shown; (b) schematic innervation pattern of *Menidia beryllina* (= *M. gracilis*), Atherinopsidae, modified from^53^; (c) schematic innervation pattern of *Polycentrus schomburgkii*, Polycentridae, modified from^13^; (d) schematic innervation pattern of Polynemidae. AbPF, *abductor profundus filamentaris*; AbPRc, *abductor profundus radialis p. ceterae*; AbSF, *abductor superficialis filamentaris*; AbSR, *abductor superficialis radialis*; AdSF, *adductor superficialis filamentaris*; AdSR, *adductor superficialis radialis*; Ana, angulo-articular; ArV, *arrector ventralis*; Asph, autosphenotic; Boc, basioccipital; Bsph, basisphenoid; Cl, cleithrum; Dt, dentary; Ecp, ectopterygoid; Enp, endopterygoid; Epoc, epioccipital; Exoc, exoccipital; F, pectoral filaments; Fr, frontal; IIIMd, *ramus mandibularis trigeminus*; IIIMx, *ramus maxillaris trigeminus*; IIIOS, *ramus ophtalmicus superficialis trigeminus*; Int, intercalar; LEth, lateral ethmoid; Me, mesethmoid; Mtp, metapterygoid (cut); Mx, maxilla; Na, nasal; OSV, compound ventral ramus of occipito-spinal nerves; Pa, parietal; Pal, palatine; Pop, preopercle (cut); Pmx, premaxilla; Pro, prootic; Psph, parasphenoid; Pst, posttemporal; Pto, pterotic; Ptsph, pterosphenoid; Qd, quadrate; R, pectoral-fin rays; Ra, retroarticular; RLA-OP, orbito-pectoralis branch of the *ramus lateralis accessorius*; Scl, supracleithrum (cut); Soc, supraoccipital; SV1–2, ventral *ramus* of spinal nerves 1–2; V, vertebra; VIH, *ramus hyomandibularis facialis*.

Four main nerve components serve the rays and filaments of the pectoral fin: compound ventral *ramus* of the occipito-spinal nerves (OSV), ventral *ramus* of the first spinal nerve (SV1), orbito-pectoral branch of the *ramus lateralis accessorius* (RLA-OP), and ventral *ramus* of the second spinal nerve (SV2; Fig. 8a). Shortly after exiting through the exoccipital foramina, two ventral *rami* of the occipito-spinal nerves (OSV1 and OSV2) fuse to each other to form a single nerve (OSV) that, subsequently, merges ventrally with the ventral *ramus* of the first spinal nerve (SV1). The resulting compound nerve (OSV + SV1) runs in between the boundaries of the *adductor superficialis radii* (dorsal) and *adductor profundus radii* (ventral), traverses the scapular foramen, and then passes to the lateral portion of the pectoral girdle (Figs. 5–7). Distally, OSV + SV1 innervates the marginal (first) ray of the pectoral fin (Fig. 8a).

The third major component involved in the innervation of the pectoral fin is the orbito-pectoral branch of the *ramus lateralis accessorius* (RLA-OP; Figs. 5–8a). This nerve is comparatively thicker than the remaining nerves serving the pectoral fin and arises from the *trigeminus-facialis* trunk, curving dorsally and running posteriorly beneath the autosphenotic and pterotic (Fig. 8a). RLA-OP then continues beyond the posterior end of the pterotic and proceeds towards the posttemporal. Medial to this bone, RLA-OP runs ventrally, passing medial to the supracleithrum and cleithrum. At the level of the dorsal region of the cleithrum, RLA-OP gives off two thin branches that innervate the middle and ventral pectoral-fin rays (Fig. 5a). Slightly after or at the same level of this branching, RLA-OP merges with the also thick ventral *ramus* of the second spinal nerve (SV2; Fig. 7), forming a massive compound nerve (RLA-OP + SV2; Figs. 5-8d). RLA-OP + SV2 is usually posterior to and much thicker than OSV + SV1, and runs over the medial surface of the *adductor superficialis fili* (Figs. 5–7). RLA-OP + SV2 then subdivides ventrally into several branches that run along the pectoral filaments (Figs. 5–7). The branches serving each filament are extremely thick compared to those serving the unmodified pectoral-fin rays (derived from OSV + SV1 and RLA-OP only; see above).

**Figure 9:**
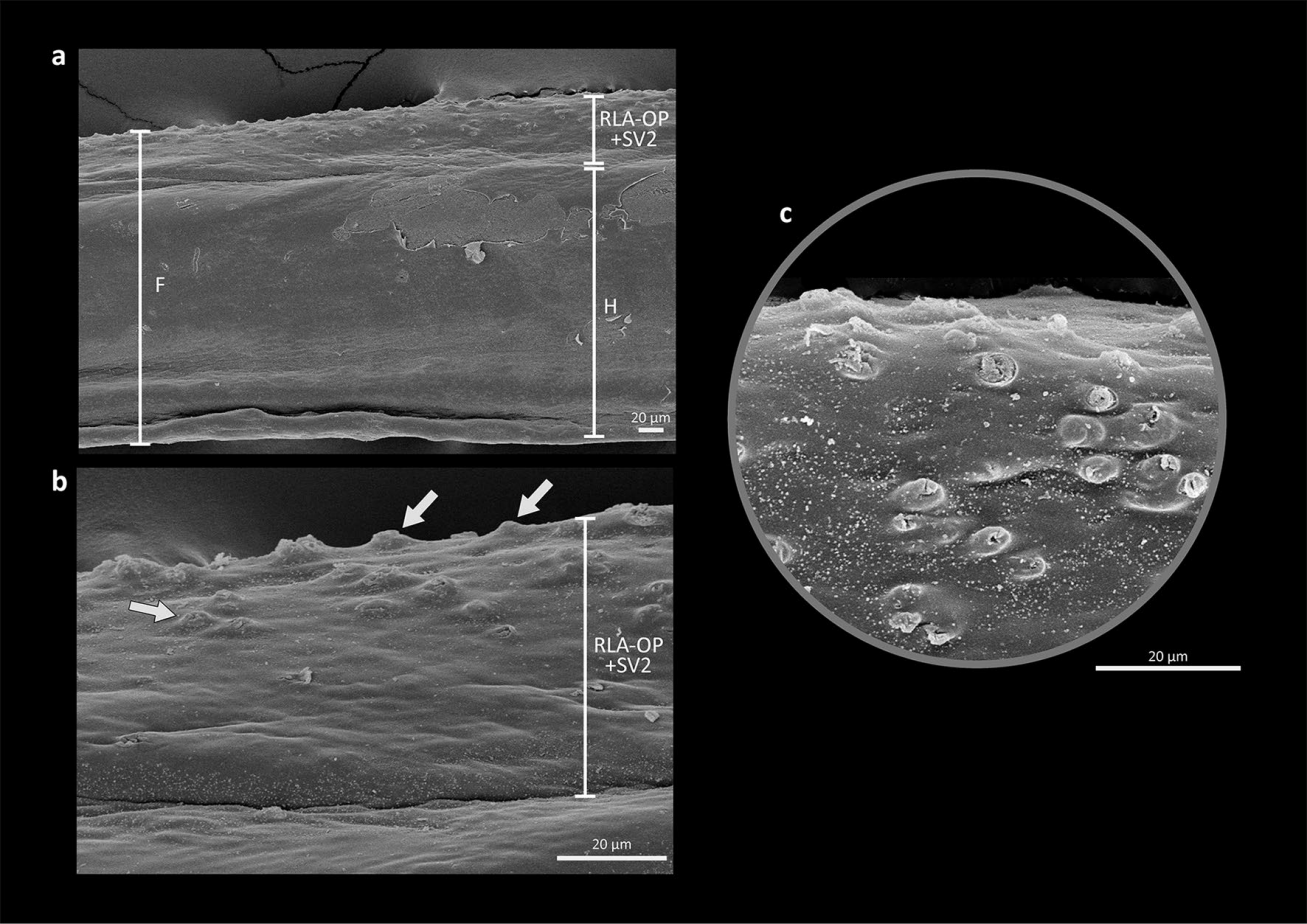
Electron microscopy of a pectoral filament of *Filimanus xanthonema*, Polynemidae. (**a**) entire pectoral filament; (**b**) detail of the latero-dorsal region of the pectoral filament, in which the RLA-OP + SV2 nerve runs; (**c**) detail of taste buds. F, pectoral filament; H, region of the pectoral filament composed only by hemitrichia; RLA-OP, orbito-pectoralis branch of the *ramus lateralis accessorius*; SV2, ventral ramus of the second spinal nerve. Arrows indicate taste buds along the filament.

Little variation in this generalized pattern is observed among different polynemid taxa. RLA-OP varies in thickness across polynemids, but is always comparatively much thicker than SV1 and OSV. In *Pentanemus*, this nerve bifurcates immediately after exiting the neurocranium, with the two resulting branches merging with SV2 at the cleithrum level, as it does in other polynemids (Fig. 5a). *Eleutheronema* is unique in having most part of RLA-OP + SV2 running posterior to the *adductor superficialis fili* (Fig. 6a). In *Pentanemus* and *Parapolynemus* the main trunk of RLA-OP + SV2 overlaps, in medial view, the main trunk of OSV + SV1 at the pectoral girdle level (Fig. 5). In all other polynemids, these two trunks are more easily distinguishable in medial view.

## Discussion

The evolution of the pectoral filaments in polynemids involves modifications in the skeleton, muscles, and nerves of the pectoral fin and girdle in comparison with the generalized pattern found in most percomorphs. In most percomorphs, all pectoral-fin rays have similar length and thickness and the pectoral radials are also similar in size (Fig. 2e). In polynemids, the pectoral filaments are formed by unbranched ventral rays that, during development, become thicker and usually much longer than the unmodified dorsal ones (Figs. 1, 2a–c). The extreme enlargements of pectoral radials 3 and 4 are other obvious distinctive osteological modifications in the polynemid pectoral fin (Figs. 1, 2a–c). Although the ventral radials are often progressively larger than the dorsal ones in many other percomorphs, this enlargement is much less pronounced than that of polynemids^18,22–35^. This specialization in polynemids is coupled with a dramatic anteroventral expansion of the posterior facet of the coracoid that articulates with radial 4. This facet is located dorsal to the postcoracoid process and its anteroventral expansion is clearly seen through ontogeny (Fig. 1), giving to the coracoid of adult polynemids a roughly hourglass shape in medial view (Fig. 2a–c). In the vast majority of other percomorphs, including those proposed to be closely related to polynemids (e.g., mugilids, pleuronectiforms, and sciaenids), the posterior facet of the coracoid is much smaller and often lacks a direct association with radial 4 (e.g., ^18,22–40^). The articulation of the third pectoral radial with the adjacent radials (2 and 4) and with the coracoid may be reinforced by interdigitations (Fig. 2a, b), a condition that is quite unusual among generalized percomorphs. The fourth radial is more prominently expanded than in other percomorphs and, in most polynemids, it is the sole element articulating with the elongate pectoral filaments (Figs. 1, 2a, b). A similar enlargement in the fourth radial is also reported for the tripod fish genus *Bathypterois* (Aulopiformes: Ipnopidae^41^). Curiously, *Bathypterois* also has pectoral-fin rays modified into elongate filaments, but its more elongated rays are the dorsal ones that articulate with pectoral radial 1^41^.

Modifications in the pectoral musculature of polynemids are even more striking. In the generalized condition for percomorphs, there are two muscles uniquely inserting onto the marginal (first) pectoral-fin ray, the *arrector ventralis* and the *arrector dorsalis*^21,31,40,42–44^. The remaining rays are served laterally by the *abductores* and medially by the *adductores* muscles^21,31,40,42–44^. The *abductores* and *adductores* are usually subdivided into outer (*superficialis*) and inner (*profundus*) layers, but never subdivided into dorsal and ventral parts. Apart from the *arrectores* muscles, the entire pectoral musculature of polynemids is divided into two independent segments: a *segmentum radii* serving the unmodified pectoral-fin rays and a *segmentum fili* serving the pectoral filaments (Figs. 3–7). The pectoral musculature of polynemids was briefly described by Kang et al.^8^, but the authors missed the existence of the deepest portion of the *adductor* muscle of the *segmentum fili*, which is herein identified as the *adductor profundus fili* (Fig. 6b). The muscle named “ventral section of *adductor profundus* (ADPV)” by Kang et al.^8^ actually is not the deepest, but rather an intermediate division of the *adductor* mass associated with the pectoral filaments and thus corresponds to our *adductor medialis fili* (Fig. 6b). Those authors also did not describe important aspects of the *adductor medialis fili* (= “ventral section of the *adductor profundus*” in their terminology), such as its differentiation into *partes endomedialis* and *ectomedialis* and the variation in the muscle insertion on the filaments. In most percomorphs, the *abductor* musculature arises solely from the cleithrum and coracoid, and, in only a few cases, small fibers also originate from the radials (e.g., *Gadus*^21^). The *abductor profundus radii* of all examined polynemids has small fibers originating from pectoral radial 3, a condition not found in any of the examined representatives of taxa proposed as possibly closer to polynemids (Mugilidae, Sciaenidae, and Sphyraenidae) based on morphology^3,8,14,45–50^. According to Winterbottom^21^ the *arrector dorsalis* is the first muscle to separate from the whole medial muscle mass during ontogeny. However, in polynemids this division is incomplete and the *arrector dorsalis* and the *adductor profundus radii* share fibers even in adults (Fig. 6b). A similar condition applies to the *adductor radialis*, which is usually fully separated from the *adductor profundus radii* in most percomorphs, but not in polynemids, which have the two muscles partially continuous.

Representatives of several scorpaeniform lineages—Apistidae, Eureniidae, Hoplichthyidae, Peristediidae, Synanceiidae, and Triglidae—have a few ventralmost pectoral rays free from an interradial membrane that, to varying degrees, are somewhat independent from the main body of the pectoral fin. These modified rays are often termed “free rays” in these scorpaeniforms, and they resemble the pectoral filaments of polynemids in some aspects. The musculature associated with the scorpaeniform free rays is variously separated from the muscles serving the remaining pectoral-fin rays^40^. However, these separations are reported to be often partial (e.g., common origin and separate insertions) and/or do not involve all pectoral muscles^40^. These conditions contrast with the complete degree of separation into *segmenta radii* and *fili* seen in polynemids. In any event, the similarities in the subdivision of the musculature of the pectoral musculature of polynemids and the noted scorpaeniforms are most parsimoniously interpreted as convergences given the large phylogenetic distance between these lineages^51,52^. The ophidiiform *Dicrolene* is another distantly related percomorph that also has ventral free rays apparently moving independently from the main body of the pectoral fin. To our knowledge, no study addressing the soft anatomy of the pectoral fin and girdle of *Dicrolene* has been published so far, preventing us from making pertinent comparisons with polynemids.

Information on the innervation of the pectoral fin of bony fishes is extremely scarce, hampering the precise identification of generalized and specialized innervation patterns in Percomorphacea. However, from what is known, the ventral *rami* of the occipito-spinal (OSV), first spinal (SV1), and second spinal (SV2) nerves are the most frequently associated with the pectoral-fin rays in the group^13,45,53–55^. Several taxa additionally have the orbito-pectoral branch of the *ramus lateralis accessorius* (RLA-OP) innervating the pectoral fin^13^. A survey of the literature indicates that these four main nerve branches have similar thickness and can combine in a multitude of different patterns to innervate the fin (Figs. 8b, c). Freihofer^13^ identified major patterns of the RLA and associated nerves for several percomorph families, and described, in detail, the morphology of these nerves for six species. He concluded that spinal innervation was very consistent among these species, with the pectoral-fin rays being innervated mainly by two or three ventral *rami* of occipito-spinal nerves (OSV) and by the first spinal nerve (SV1), arriving in the pectoral fin via two branches. Both OSV and SV1 can share fibers with the RLA branch, if present (Fig. 8c). The compound branch OSV + RLA-OP normally innervates the dorsal pectoral-fin rays, while SV1 + RLA-OP frequently arrive at the middle and ventral pectoral-fin rays (Fig. 8c).

Polynemids have the main trunks of SV1 and OSV fusing medially at the pectoral girdle and, contrastingly to the pattern described by Freihofer^13^ for several percomorphs, also have the second spinal nerve (SV2) arriving at the pectoral girdle (Figs. 5–7, 8a, d). Although in *Menidia* OSV + SV1 are indeed bound together and SV2 also participates on the pectoral girdle innervation (Fig. 8b;^53^), threadfins differ by having SV2 remarkably thicker than SV1 and OSV nerves (Figs. 8a, d) and receiving all fibers from the RLA-OP branch, a condition not reported elsewhere. Prior to its fusion with SV2, RLA-OP emits small branches that dive into the middle and ventral pectoral-fin rays, whereas the compound RLA-OP + SV2 nerve goes straight to the pectoral filaments, traversing through the entire length of each filament (Figs. 5–7, 8a, d). The distinctive innervation pattern in the pectoral fin of polynemids has important functional implications.

According to Freihofer^13^ the RLA comprises “nerves whose cell bodies are located in the geniculate ganglion and whose distal fibers extend to the parietal region of the head, (…) trunk, (…), fins (…) and innervate terminal buds, that is, taste buds located on the external surface of the head and body exclusive of the snout”. He also pointed out that the main branches of the RLA carry only gustatory/taste fibers. As described above, the polynemid RLA-OP + SV2 branches run along the entire length of each pectoral filament, and small protuberances with an external opening that clearly correspond to terminal buds^56–58^ were visible through scanning electron microscopy on the latero-dorsal surface region of the filament of some examined species (Fig. 9). The presence of these buds and the innervation by RLA-OP indicates that the pectoral filaments of polynemids have gustatory function. SV2, in turn, should provide the general tactile function. Judging by the extremely large diameter of the branches of RLA-OP + SV2 serving each pectoral filament, these structures should be highly sensitive to these two types of stimuli. The pectoral filaments have been vaguely reported in the literature to act as a “sense organ”, with no explicit definition of which types of senses would be involved^1^. Kang et al.^8^ described that “two nerves were found fused with each other and running most of the length of each filament-like ray (…) support[ing] the likely tactile or gustatory function of each ray". However, they neither identified those nerves nor provide any evidence supporting the suggested sensorial functions. By identifying the nerve components serving the pectoral filaments and discovering their terminal buds, our study is the first to demonstrate that these structures have both gustatory and tactile function.

Some gadiforms and ophidiiforms also respond to taste stimuli through modified rays of the pelvic or dorsal fins^59–61^. These structures are also innervated by branches of the RLA. Via a series of ex situ experiments, Herrick^60^ demonstrated that both pelvic-fin and dorsal-fin free rays of some gadiforms are very reactive to gustatory stimuli and clearly take part in their foraging behaviour. Some authors suggested that these highly developed gustatory capabilities in modified fins represent adaptations for the benthic and/or nocturnal feeding habits^59,60^. This conclusion matches the behaviour of polynemids. Threadfins usually live in turbid and muddy waters and actively search for food by touching the pectoral filaments over the sea bed, which are forward positioned to maximize the efficiency of food exploration^1^.

As noted above, triglids are among the rare taxa that have free pectoral-fin rays resembling the polynemid pectoral filaments. Even though triglids have subdivided pectoral musculature serving the dorsal pectoral-fin rays and the few ventral free rays, they lack an RLA branch or any other branch of *trigeminus-facialis* trunk supplying the pectoral girdle^13^. As a consequence, their free rays most likely have only tactile and locomotor functions^60,62^. In triglids the distal tip of each free ray is full of ridges and papillae, ending in a terminal knob^63^, but not bearing any terminal bud^60,62,64^.

## Conclusions

The separation and differentiation of the pectoral filaments from the unmodified pectoral fin in polynemids is coupled with several changes in the skeletal development. Pectoral radial 3 undergoes a drastic enlargement, a shift in its position, and a loss of its articulation with all or most fin rays or filaments. Pectoral radial 4 similarly expands ventrally during ontogeny and, in adults, articulates solely with the pectoral filaments. The posterior articular facet of the coracoid greatly expands anteroventrally, so that the bone assumes an hourglass shape in adults. Compared to generalized percomorphs, adult polynemids more than double the number of independent divisions of the intrinsic pectoral musculature. The *adductor* and *abductor* muscles masses are divided into a *segmentum radii* serving the unmodified fin, and a *segmentum fili* serving the pectoral filaments. The *adductor profundus radii* shares fibers with the *arrector dorsalis* and the *adductor radialis*, two muscles typically separated from the whole medial musculature in other percomorphs. Innervation of the pectoral filaments by massive components of the second spinal nerve and of the *ramus lateralis accessorius* indicate that the pectoral filaments have both tactile and gustatory functions, a conclusion additionally corroborated by the finding of taste bud on the surface of the pectoral filament.

## Data Availability

All data generated or analysed during this study are included in this published article.
